# Self-Relevance Appraisal Influences Facial Reactions to Emotional Body Expressions

**DOI:** 10.1371/journal.pone.0055885

**Published:** 2013-02-06

**Authors:** Julie Grèzes, Léonor Philip, Michèle Chadwick, Guillaume Dezecache, Robert Soussignan, Laurence Conty

**Affiliations:** 1 Laboratoire de Neurosciences Cognitives (LNC) - INSERM U960 & IEC - Ecole Normale Supérieure (ENS), 75005 Paris, France; 2 Centre des Sciences du Goût et de l'Alimentation (CSGA) UMR 6265 CNRS - 1324 INRA, Université de Bourgogne, 21000 Dijon, France; 3 Laboratoire de Psychopathologie and Neuropsychologie (LPN, EA2027), Université Paris 8, Saint-Denis 93526 cedex, France; University of Bologna, Italy

## Abstract

People display facial reactions when exposed to others' emotional expressions, but exactly what mechanism mediates these facial reactions remains a debated issue. In this study, we manipulated two critical perceptual features that contribute to determining the significance of others' emotional expressions: the direction of attention (toward or away from the observer) and the intensity of the emotional display. Electromyographic activity over the *corrugator* muscle was recorded while participants observed videos of neutral to angry body expressions. Self-directed bodies induced greater *corrugator* activity than other-directed bodies; additionally *corrugator* activity was only influenced by the intensity of anger expresssed by self-directed bodies. These data support the hypothesis that rapid facial reactions are the outcome of self-relevant emotional processing.

## Introduction

Emotional expressions are critical to the coordination of social interactions by providing information about the emitter's emotional states and behavioral intentions and by evoking reactions in the observer [1]–[4]. The research agrees that when exposed to emotional expressions, people display rapid facial reactions (RFRs) detectable by electromyography (EMG) [5]–[9]. While viewing static or dynamic happy faces elicits increased *zygomaticus major* activity (pulling the corners of the mouth back and upwards into a smile), angry faces evoke increased *corrugator supercilii* activity (pulling the brows together) [10]–[20]. Nevertheless, exactly what mechanism mediates these facial reactions remains a debated issue [8], [21]–[23].

One major theoretical framework proposes that these facial reactions reflect the readout of emotional processing [6], [24], [25]. Within this framework, the appraisal perspective postulates that a multimodal organization of response patterns (which includes facial expressions and physiological reactions) is established according to appraisal configurations (novelty, coping potential, relevance, etc.) that are emotion-specific [1], [26], [27]. The emotional readout framework implies that people would be disposed to react with emotion-specific response patterns to biologically relevant stimuli such as expressions of anger [6]; and also that a given facial expression can elicit a different emotion and thus a divergent reaction in the observer, such as, for instance, a posture of submission in response to a threatening expression. This partly explains why facial reactions are less automatic than first thought [28], and why their production varies substantially as a function of the social context, the perceived emotion [29], [30], and the relationship between the expresser and the observer [31].

The present experiment manipulated the self-relevance of stimuli to further verify the contribution of affective processes to RFRs. Recent work converges toward the view that the ability to initiate adapted behaviors in response to others' emotional signals mainly depends on the capacity to correctly evaluate the functional significance of the emitted signal for the self [32]. Several factors can therefore influence how self-relevant a given emotional signal is, thereby determining how an observer will evaluate and respond to it. Direction of gaze and body posture are among the most socially relevant cues through which we gain information regarding the source of an individual's emotional reaction and the target of their impending actions. Such cues are particularly significant for anger because of their prime importance in regulating social interactions in both human [33] and non-human [34] primates. Facial expressions of anger have been shown to be more accurately and quickly recognized, and judged to be more intense, when coupled with direct gaze [35]–[40]. Additionally, Hess et al. [31] revealed an increase in the EMG activity of the *orbicularis occuli* in response to funny films of increasing intensity in the presence of friends but not of strangers; strongly suggesting that both self-relevance appraisal and the intensity of eliciting stimuli are important determinants of emotional facial reactions.

Here we elaborated upon the above-mentioned results by varying two independent critical cues in face to face interactions: body orientation, proven to be important in determining to whom social attention is directed (toward or away from the observer), and the intensity of the emotional display (different levels of angry body expressions). We presented dynamic bodily expressions of anger of increasing intensity, directed toward or away from the observer. First, whether previous findings could be generalized to angry body expressions remains to be established, but if affective processes participate in facial reactions, RFRs should be elicited for other forms of emotional communication signals than facial expressions, such as bodily expressions. Second, the observer's facial EMG responses to emotional expressions as a function of face direction has only been explored in two studies [17], [41]. Besides presenting conflicting results, these studies were limited in that subjects were explicitely instructed to determine the presence or absence of eye contact. Thus, by potentially influencing the importance attributed to gaze direction, they might have biased facial EMG activity. Yet, if the relevance of other's emotional expressions impact the oberver's affective processing, being the target of an expression of anger is expected to *implicitely* trigger more activity in the *corrugator supercilii*, as compared to being a simple observer of that expression. Moreover, the level of muscle activity is expected to fluctuate with the intensity of the displayed expression.

## Methods

### Ethics

The present study obtained ethics approval from the local research ethics committees (CPP Ile de France III and Institut Mutualiste Montsouris) at all institutions where participants were recruited and human experimentation was conducted.

### Stimuli

Eight professional actors (four males) were hired and instructed to begin at neutral and to increase their expression of anger in seven to nine 3 s increments according to the experimenters signal in front of a camera until deemed satisfactory. Performances were filmed with two cameras: one was facing the actor; the second at a 45°angle relative to the first creating the impression that the expression was aimed toward the observer (oriented-to-self condition) or toward another (oriented-to-other condition).

Videos were edited using *Windows Movie Maker* and several 2 sec (25 frames per second) fragments were selected to obtain two extracts for each condition from neutral to extreme anger with two different viewpoints. Clips of actors seen from the side were flipped to obtain equal numbers of left and right videos and faces were blurred using the *Adobe After-effect* software, to preclude extraction of any emotional cues conveyed by them and restricting information to the body.

**Figure 1:**
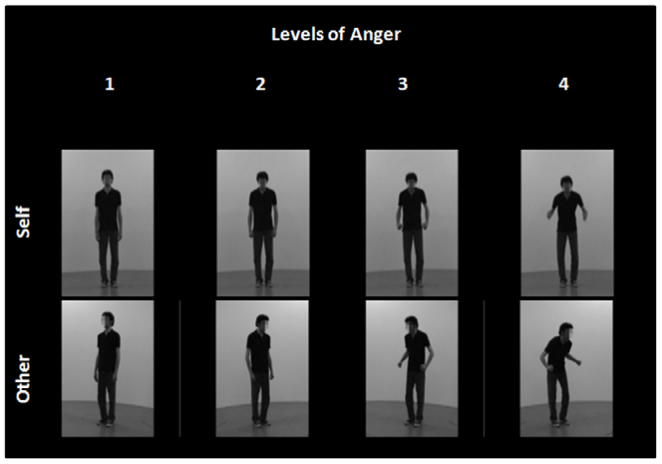
2*4 factorial design. Short movies of neutral (1), mild (2), moderate (3) and intense anger (4) oriented-to-Self and oriented-to-Other were presented.

Selection of the final material was based on the results of a behavioral pilot study. A total of 312 edited video clips including all the original steps from neutral to anger for each actor were presented on a PC screen. Participants (n = 23) were instructed to evaluate the intensity of the actor's bodily expression on a continuous scale from neutral to high anger. Two-tailed paired t-tests were used to compare increments and the results permitted the selection of the most consistently convincing performances of each actor's range, corresponding to 4 significantly different steps in the degree of expressed anger (p<0.05). We retained 96 videos corresponding to 8 actors, 4 levels of anger (neutral; mild; moderate; intense anger) and 2 points of view (oriented to self and other, both right and left viewpoints). A 2×4 factorial design was built, with Target of Attention (Self or Other) and Levels of emotion (neutral (1); mild (2), moderate (3) and intense anger (4)) as factors (**see** **Fig. 1**).

### Validation of the stimuli

Two behavioral experiments were conducted on the selected 96 stimuli.

#### Identification of Anger

This study assessed the ability to identify anger from dynamic body expressions. Participants (n = 20) were requested to decide (forced-choice) for each video whether the expression of the actor was “neutral”, “angry” or “other”. The order of the stimuli was fully randomized, as well as the order of the response words on the response screen. Categorization rates were percent transformed and submitted to a repeated measures ANOVA with within-subject factors of Target of Attention (Self or Other), Levels of Emotion (1, 2, 3, 4) and Choice (Anger, Neutral, Other). Greenhouse-Geisser epsilons (ε) and p values after correction were reported where appropriate. Post-hoc comparisons (two-tailed t-tests) were performed for the analysis of simple main effects when significant interactions were obtained.

**Figure 2:**
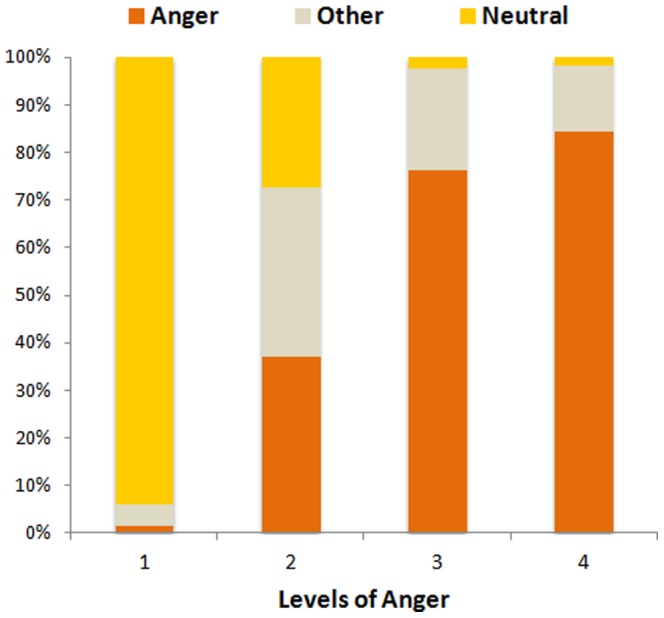
Results from the categorization task. Mean percentage for each choice (Anger, Neutral or Other) of the categorization task plotted as a function of the Levels of Emotion (1, 2, 3, 4).

The ANOVA revealed a main effect of Choice, F(2,38) = 36.57; *p*<.001, but no main effect of Target (F(1,19) = 1.30; *p* = .26), nor a main effect of Levels of Emotion, F(3,57) = 2.42; *p* = .075; ε = 0.67; *p_corr_* = .101. Of interest, only the interaction between Levels of Emotion and Choice, F(6,114) = 143.06; *p*<.001; ε = 0.55; *p_corr_*<.001 reached significance. For both Self- and Other-directed expressions, level 1 was correctly categorized as “Neutral” (as compared to “Anger” and “Other”, all *ps*<.001), and levels 3 and 4 as “Anger” (as compared to “Neutral” and “Other”, all *ps*<.001). The response accuracy for these conditions was above 75% and differed from chance level (33%) at p<0.001 (**See** **Fig. 2**). This was not the case for the mild levels of anger where accuracy did not significantly differ from chance level (Other2 = 36%, p = .497; Self2 = 39%, p = .195). These mild levels were ambiguous as participants responded “Neutral”, “Angry” or “Other” equally for both Self- and Other-directed expressions (all *ps*>.169; **See** **Fig. 2** **and Table S1**).

#### Subjective Feelings

The second experiment assessed the intensity of the participants (n = 20)’ feelings when confronted with angry body expressions. Participants were requested to evaluate the intensity of Felt Confusion, Surprise, Sadness, Threat and Irritation on 5 graduated scales from 0 to 9. The five scales appeared on the screen following each video, and their order was randomized between subjects. The order of the stimuli was fully randomized. Ratings were submitted to a repeated-measures ANOVA with within-subject factors of Feelings (Confusion, Surprise, Sadness, Threat and Irritation), Target of Attention (Self or Other) and Levels of Emotion (1, 2, 3, 4). Greenhouse-Geisser epsilons (ε) and p values after correction were reported where appropriate. Post-hoc comparisons (two-tailed t-tests) were performed for the analysis of simple main effects when significant interactions were obtained.

**Figure 3:**
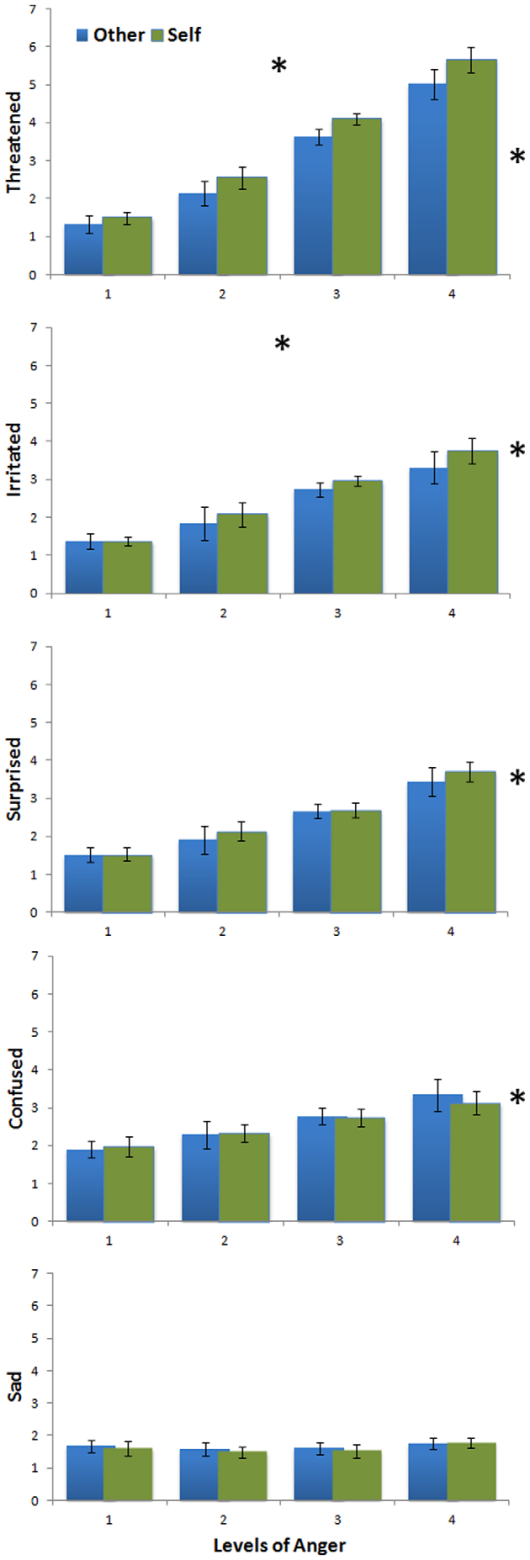
Intensity of felt emotions. The intensity of Felt Emotions (Threatened, Irritated, Surprised, Confused and Sad) with standard-errors are plotted as a function of the Target of Attention (S for Self, O for Other), and the Levels of Emotion (1, 2, 3, 4). The grey asterisks on the right signal feelings that significantly increased with Levels of Emotion. Blacks asterisks on panels signals feelings that significantly increased for Self as compared to Other-directed body.

The ANOVA indicated a main effect of Feelings, F(4,76) = 16.09; *p*<.001, ε = 0.82; *p_corr_*<.001, and a main effect of Levels of Emotion, F(3,57) = 48.59; *p*<.001; ε = 0.38; *p_corr_*<.001, but no main effect of Target, F(1,19) = 2.64; *p* = .12. There was a significant interaction between Feelings * Levels of Emotion, F(12,228) = 19.57; *p*<.001; ε = 0.37; *p_corr_*<.001. The intensity of the Feelings increased with the Levels of Emotion (Level1<Level2<Level3<Level4 - all t(19)>36.22; all *ps*<.001), except for Sadness (Level1 = Level2 = Level3<Level4)(**See Table S2 and** **Fig. 3**). Of interest here, there was a significant interaction between Feelings * Target, F(4,76) = 6.25; *p*<.001; ε = 0.68; *p_corr_* = .001. Self- as compared to Other-directed expressions were perceived as more Threatening (t(19) = 2.67; *p* = .015) and more Irritating (t(19) = 2.54; *p* = .02). There was no difference for the other Feelings (ps>.23).

We then conducted a repeated-measures ANOVA with within-subject factors of Feelings (Threat and Irritation), Target of Attention (Self or Other) and Levels of Emotion (1, 2, 3, 4). This ANOVA revealed a main effect of Feelings, F(1,19) = 14.63, *p* = .001: participants felt more threatened than irritated when confronted with body expressions of anger (Mean (SEM) Threat = 3.24(.19); Irritation = 2.42(.23)(See Figure 3). It also revealed a main effect of Target, F(1,19) = 7.84; *p* = .011; a main effect of Levels of Emotion, F(3,57) = 65.51; *p*<.001; ε = 0.42; *p_corr_*<.001, a significant interaction between Feelings * Levels of Emotion, F(3,57) = 14.86; *p*<.001; ε = 0.55; *p_corr_*<.001, a significant interaction between Feelings * Target, F(3,57) = 19.87; *p*<.001 but no triple interaction between Feelings * Levels of Emotion * Target, F(3,57) = .082; *p* = .97. Importantly here, while participants rated their feeling of both Threat and Irritation higher when exposed to Self- as compared to Other-directed expressions and increased their rating of intensity of feeling as a function of the increased intensity of the stimuli, these effects were more marked for feelings of Threat (**see table S2**). Together, these results strongly suggest that the perception of Self-directed angry body expressions mainly prompted a feeling of Threat in the observer, as compared to other feelings (**See** **Fig. 3**).

### Facial EMG experiment

#### Participants

Forty-four participants (21 women) participated in the physiological experiment. All had normal or corrected-to-normal vision, were right-handed, naive to the aim of the experiment and presented no neurological or psychiatric history. All provided written informed consent according to institutional guidelines of the local research ethics committee and were paid for their participation. Due to a bad signal-to-noise ratio in physiological signals, four subjects (2 men) were excluded from final analysis leaving 40 participants (mean age = 24±0.4 years).

#### Experiment

Participants had to fix a white cross centered on a 19-inch black LCD screen for a random duration of 800 to 1200 ms followed by a silent 2000 ms video showing an actor in one of the eight experimental conditions. Each video was followed by an inter-stimulus interval of 1000 ms. Additionally, 15 oddball stimuli (upside-down video-clips; see below) and 38 null events (black screen of 2 sec) were included pseudo-randomly within the stimulus sequence. The order of the stimuli was fully randomized. Subjects were instructed to press a button each time the upside-down video-clip appeared to ensure they paid attention to all the stimuli throughout the session. The participants performed at 100% of accuracy (at mean 648±22 ms) in this oddball task.

#### Data acquisition and analysis

Using the acquisition system ADInstruments (ML870/Powerlab 8/30), EMG activity was continuously recorded using Sensormedics 4 mm shielded Ag/AgCl miniature electrodes (Biopac Systems, Inc). Fixation cross and stimuli onset were automatically signaled on the channels of the LabChart Pro software by a PCMCIA Parallel Card (Quatech SPP-100). Before attaching the electrodes, the target sites of the left face were cleaned with alcohol and gently rubbed to reduce inter-electrode impedance. Two pairs of electrodes filled with electrolyte gel were placed on the target sites and secured using adhesive collars and sticky tape. Following the guidelines proposed by Fridlund & Cacioppo [42], the two electrodes of a pair were placed at a distance of approximately 1.5 cm over 2 muscle regions associated with different emotional expressions. Activity over the left *corrugator supercilii* muscle, which lowers brows, was used as a marker of negative emotional expression [6]. Activity over the left *zygomaticus major* muscle, which pulls lip corners up and indexes pleasure/happiness, was used as a control recording site to verify that participants responded selectively to anger expressions. The ground electrode was placed on the upper right forehead. The signal was amplified, band-pass filtered online between 10–500 Hz, and then integrated. EMG trials containing artifacts were manually rejected. No more than 15% of the trials were dropped per muscles. Integral values were subsampled offline at 10 Hz and log transformed to reduce the impact of extreme values [9], [23]. Values were then standardized within participants and within muscle to allow comparisons. Temporal profiles of facial EMG during the first 1000 ms following stimulus onset were investigated by calculating mean amplitudes during 10 time intervals of 100 ms. Pre-stimulus values (computed over 200 ms before the stimuli onset) were then subtracted from post-stimulus activity to measure the activity level caused by viewing each stimulus (i.e., to calculate the change from baseline). EMG activity was thus defined as the change from the baseline occurring between 0 and 1000 ms after stimuli onset [10], [23]. Finally, mean levels of corrugator and zygomaticus activity were computed separately for each experimental condition.

Physiological data were first submitted, separately for each muscle, to repeated measures ANOVA using Target of Attention (Self or Other), Levels of Emotion (1, 2, 3, 4) and Time Windows (10) as within-subject factors. Second, when the Time Windows factor interacted with another factor of interest, we performed post-hoc t-tests to determine the time windows for which the effect occurred and submitted the mean activity of these windows to a new ANOVA using Target of Attention (Self or Other) and Levels of Emotion (1, 2, 3, 4) as within-subject factors. Greenhouse-Geisser epsilons (ε) and p values after correction were reported when appropriate. Post-hoc comparisons (two-tailed t-tests) were also performed for the analysis of simple main effects when significant interactions were obtained.

## Results

### Corrugator activit

**Figure 4:**
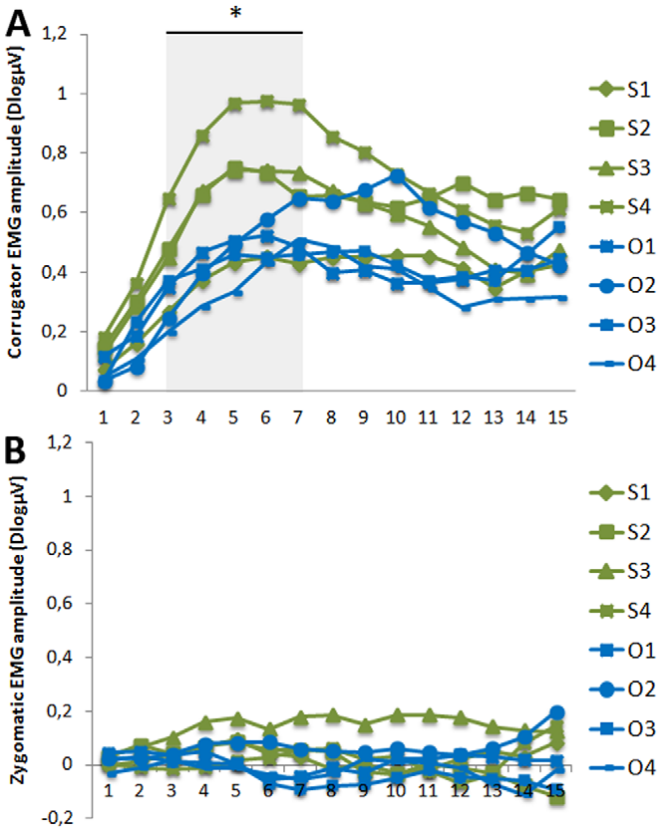
Time course of the mean EMG activity. A) Over the *corrugator supercilii* region as a function of the Target of Attention (S for Self (green), O for Other (blue)) and the Levels of Emotion (1,2,3,4). Activity reflects average activation during each 100-ms time interval. B) Over the *zygomaticus* region as a function of the Target of Attention (S for Self, O for Other) and the Levels of Emotion (1,2,3,4).

The ANOVA indicated significant effects of Target of Attention, F(1,39) = 11.05; *p* = .002, Levels of Emotion, F(3,117) = 2.71; *p* = .048, and Time Windows F(9,351) = 45.55; *p*<.001; ε = 0.20; *p_corr_*<.001 (**See Table S3, and** **Fig. 4**). The interaction between Target of Attention and Levels of Emotion, F(3,117) = 5.39; *p* = .002; ε = 0.77; *p_corr_* = .004, was significant after correction, whereas the other interactions didn't reach significance after correction: Time Windows×Target of Attention, F(9,351) = 2.63; *p* = 0.006; ε = 0.26; *p_corr_* = .068, and Time Windows×Levels of Emotion, F(27,1053) = 1.66; *p* = .019; ε = 0.23; *p_corr_* = .127. Yet, the triple interaction between Time windows, Target of Attention and Levels of Emotion reached significance after correction, F(27,1053) = 1.67; *p*<.001; ε = 0.23; *p_corr_* = .035. We then submitted the data for each time window to a second ANOVA with within-subject factors of Target of Attention (Self or Other) and Levels of Emotion (1, 2, 3, 4). This analysis revealed that the interaction between Target of Attention and Levels of Emotion was significant between 300 and 700 ms Time windows, all F(3,117)>4.4; all *p_corr_*<.01.

**Figure 5:**
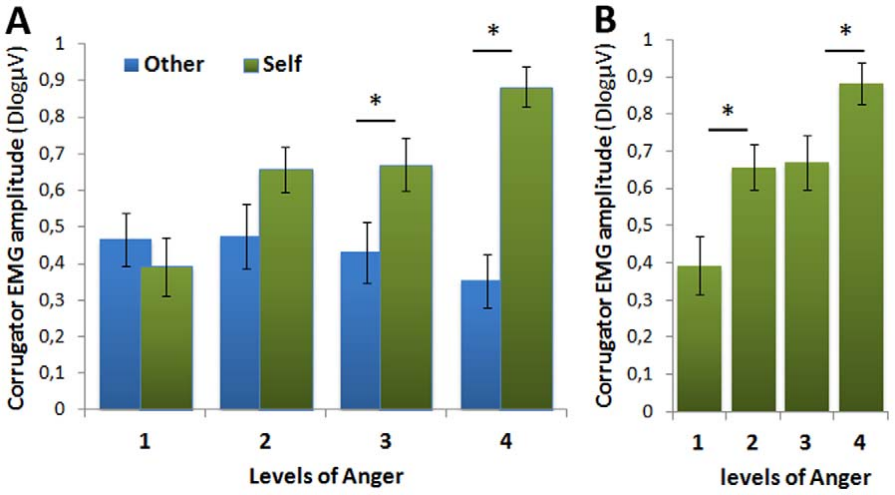
Mean activity over the *corrugator supercilii* region between 300 and 700 ms. The mean (SEM) activity is represented as a function of A) the Target of Attention (Self (green), Other (blue)) and the Levels of Emotion (1,2,3,4) and, B) only for Self-oriented conditions for the 4 Levels of Anger. *p<0.05.

We thus computed the mean activity between 300 and 700 ms and submitted these data to a second ANOVA with within-subject factors of Target of Attention (Self or Other) and Levels of Emotion (1, 2, 3, 4)(**See Table S4, and** **Fig. 5**). This second ANOVA revealed a main effect of Target of Attention. Self-directed body induced greater corrugator activity than Other-directed bodies, F(1,39) = 13.02; p<.001. An interaction between Target of Attention and Levels of Emotion was also observed, F(3,117) = 6.31; p<.001; ε = 0.75; pcorr = .002, revealing that the effect of Target of Attention increased with the Levels of Emotion: the effect of Target of Attention was not significant at level 1 (i.e. Neutral stimuli-t(39) = −.605; p = .548); failed to reach significance at level 2, t(39) = 1.855; p = .071; appeared significant at level 3, t(39) = 2.338; p = .025, and reached high significance at level 4 of emotion, t(39) = 5.826; p<.001. Interestingly, for Self-directed bodies, level 1 was significantly different from level 2 (t(39) = −2.687; p = .011); level 2 and level 3 were not significantly different, t(39) = −.134; p = .897; but level 3 appeared significantly different from level 4, t(39) = −2.342; p = .024. By contrast, the different levels of emotion did not significantly differ in the Other-directed condition, all ps>.434. Finally, post-hoc analyses revealed that activity between 300–700 ms significantly differs from 0 in all experimental conditions (all t(39)>4.7;all p<.001) suggesting that all conditions triggered RFRs (**see** **Fig. 5**).

### Zygomatic activity

Using *zygomatic* activity as a control recording site, the ANOVA with Target of Attention (Self or Other), Levels of Emotion (1,2,3,4) and Time Windows of analyses (10), as within-subject factors, did not reveal any main effect nor significant interaction, all F<1.45 (**Table S5,** **Fig. 4**).

## Discussion

Previous EMG experiments have consistently demonstrated that people tend to produce facial reactions when looking at other's facial expressions of emotion. Here, we found that participants displayed early facial reactions to body expressions of anger, as revealed by an increase of corrugator activity occurring 300 to 700 ms after stimulus onset. RFRs were stronger when anger signals were directed toward them as compared to averted gaze, and the higher the intensity of displayed anger, the stronger their facial reactions. We propose early RFRs to body expressions of anger might be related to an emotional appraisal process [38].

Our data reveal the same influence of the direction of attention in the RFRs to body expressions, as has been shown for faces [35], [38]. Using virtual avatars and manipulating face orientation, Schrammel et al. [17,17] demonstrated significantly higher *corrugator* activity for angry faces with direct gaze as compared to angry faces with averted gaze. More recently, we further provided facial EMG evidence of the critical role of attention on interpersonal facial matching by manipulating gaze direction rather than face orientation [43]. Here, even in the absence of gaze information, self-directed body expressions of anger triggered higher corrugator reactivity as compared to other-directed bodies. Our data converge with the appraisal perspective which proposes that the evaluation of emotional stimuli depends on the degree of self-relevance of the event. Within such a framework, it is proposed that anger should be rated as more intense when coupled with direct gaze as it signals a direct threat for the observer [38], [40], [44]. Indeed, this was confirmed by our behavioural pre-tests revealing that the perception of self-directed angry body expressions specifically increased the subjective feelings of being threatened.

Crucially, we have demonstrated for the first time that the intensity of bodily expressions of anger displayed by a congener enhanced RFRs only when directed toward the self. The absence of such an increase for averted bodies dismisses the possibility that these findings are strictly related to the amount of movement involved in body expressions. Together with the findings of Hess et al. [31] of increased EMG reactivity to funny films of increasing intensity in the presence of friends only, our results imply that it is the interaction between these factors that influences how self-relevant an emitted signal is and determines the levels of RFRs (here: direction of the emitters' attention and the intensity of their expression), rather than each factor individually. Moreover, our results strongly suggest that the higher the potential for interaction with another (positive in Hess et al., negative here), the higher the facial reactions in the observer.

Recently, using EEG under fMRI, we revealed that the degree of potential social interaction with another relies on the binding of self-relevant social cues 200 ms after stimulus onset in motor-related areas [45]. The present early RFRs, beginning at 300 ms after stimulus onset, may thus reflect the emotional motor response to being threatened. Activity in the *corrugator supercilii* muscle is largely accepted as a reflection of negative emotional reactions to negative-valenced stimuli, such as spiders and snakes [6], unpleasant scenes [46] or to negative facial expressions [23], [30], [47], and has also been demonstrated in response to static body expressions of fear [48], [49]. The present activity in the *corrugator supercilii muscle* triggered in response to body expressions of anger may thus relate to the observer's negative emotional reaction. As anger displays are appraised as power and dominance signals, which have been shown to trigger divergent rather than convergent responses [50], one can speculate that these RFRs convey a divergent fear response [23], [30].

RFRs over the corrugator muscle occur in response to body expressions in the absence of facial information, and regardless of body orientation and of emotional content. Although it is acknowledged that RFRs may result from multiple processes [18], [23], the presence of early RFRs in absence of facial expressions cannot be explained by strict motor mimicry as the body expressions here did not provide the cues necessary for facial motor matching. A strict motor mimicry process is indeed not sufficient to explain why RFRs are displayed to non-facial and non-social emotional pictures [6], emotional body expressions [48], [49] and auditory stimuli [51]–[53], nor why they are occasionally incongruent with the attended signals [23]. Moreover, our results are clearly at odds with the predictions that can be derived from a motor mimicry perspective, i.e. that participants should either display congruent RFRs to others' angry faces, irrespective of the direction of attention of the emitter [28] or display less mimicry when directed at the observer as anger conveys non-ambiguous signals of non-affiliative intentions [29], [37]. Yet, the present early RFRs elicited in all experimental conditions, including neutral bodies (Level 1), also rule out the possibility that they reflect emotional processes only and suggest that RFRs could partly result from a mere orienting response to the apparition of the stimuli and/or the observer's cognitive effort [54] to decode an emotional expression in the absence of facial information and/or the appraisal of goal-obstructiveness [54], [55]. Also, as the present findings were revealed using body expressions of anger only, we cannot simply rule out that motor-mimicry processes would occur under other experimental circumstances, nor specify how motor, emotional and appraisal processes might interact. Further experiments are thus needed to determine whether the present results can be generalized to a wider range of emotions as well as whether (and to what extent) both motor and affective processes operate when facial information is available [18].

To conclude, we not only demonstrate that the *corrugator supercilii* muscle can be triggered in response to angry expressions but extend these findings to dynamic bodies. The present findings corroborate the emotional readout framework and further suggest that rapid facial reactions reflect the appraisal of the context and its self-relevance which varies as a function of the emitter's direction of attention and the intensity of his/her anger.

## Supporting Information

Table S1 **Mean (SEM) recognition rate.**
(DOC)

Table S2 **Mean (SEM) intensity ratings of feelings**
(DOC)

Table S3 **Mean (SEM) data from the** ***Corrugator*** **activity submitted to a repeated measures ANOVA using Target of Attention (Self or Other), Level of Emotion (1, 2, 3, 4) and Time Windows (10) as within-subject factors.**
(DOC)

Table S4 **Mean activity (SEM) between 300 and 700 ms for the** ***Corrugator*** **muscle region submitted to a repeated measures ANOVA with within-subject factors of Target of Attention (Self or Other) and Level of Emotion (1, 2, 3, 4).**
(DOC)

Table S5 **Mean (SEM) data from the** ***zygomatic*** **activity submitted to a repeated measures ANOVA using Target of Attention (Self or Other), Level of Emotion (1, 2, 3, 4) and Time Windows (10) as within-subject factors.**
(DOC)
