# Supplementary material for: Self-Relevance Appraisal Influences Facial Reactions to Emotional Body Expressions
Source: PLoS One. 2013 Feb 6;8(2):e55885. doi: 10.1371/journal.pone.0055885 (PMC3566069; doi:10.1371/journal.pone.0055885)
Supplement: Table S1 — Mean (SEM) recognition rate. (DOC) [file pone.0055885.s001.doc]

|  | Self | | | | | | | | Other | | | | | | | |
| --- | --- | --- | --- | --- | --- | --- | --- | --- | --- | --- | --- | --- | --- | --- | --- | --- |
|  | Level1 | | Level2 | | Level3 | | Level4 | | Level1 | | Level2 | | Level3 | | Level4 | |
|  | Mean | SEM | Mean | SEM | Mean | SEM | Mean | SEM | Mean | SEM | Mean | SEM | Mean | SEM | Mean | SEM |
| Other | 3.21 | 1.28 | 36.11 | 6.28 | 25.91 | 5.92 | 15.16 | 3.38 | 5.71 | 1.71 | 37.84 | 7.76 | 18.20 | 3.80 | 14.50 | 2.50 |
| Anger | 1.71 | 1.20 | 39.59 | 4.90 | 75.05 | 5.94 | 90.82 | 3.08 | 1.25 | 0.86 | 36.73 | 5.39 | 83.95 | 4.46 | 90.14 | 4.07 |
| Neutral | 98.80 | 3.76 | 28.91 | 3.93 | 2.68 | 1.23 | 1.25 | 0.86 | 93.04 | 1.71 | 27.86 | 4.25 | 2.59 | 1.19 | 2.59 | 1.19 |
