# Supplementary material for: Self-Relevance Appraisal Influences Facial Reactions to Emotional Body Expressions
Source: PLoS One. 2013 Feb 6;8(2):e55885. doi: 10.1371/journal.pone.0055885 (PMC3566069; doi:10.1371/journal.pone.0055885)
Supplement: Table S2 — Mean (SEM) intensity ratings of feelings (DOC) [file pone.0055885.s002.doc]

|  | Self | | | | | | | | Other | | | | | | | |
| --- | --- | --- | --- | --- | --- | --- | --- | --- | --- | --- | --- | --- | --- | --- | --- | --- |
|  | Level1 | | Level2 | | Level3 | | Level4 | | Level1 | | Level2 | | Level3 | | Level4 | |
|  | Mean | SEM | Mean | SEM | Mean | SEM | Mean | SEM | Mean | SEM | Mean | SEM | Mean | SEM | Mean | SEM |
| Confusion | 1.98 | 0.26 | 2.33 | 0.22 | 2.74 | 0.24 | 3.13 | 0.37 | 1.90 | 0.22 | 2.29 | 0.22 | 2.78 | 0.30 | 3.34 | 0.42 |
| Irritation | 1.37 | 0.12 | 2.08 | 0.21 | 2.96 | 0.31 | 3.74 | 0.44 | 1.37 | 0.12 | 1.83 | 0.19 | 2.73 | 0.34 | 3.31 | 0.43 |
| Sadness | 1.60 | 0.23 | 1.49 | 0.18 | 1.52 | 0.17 | 1.78 | 0.19 | 1.68 | 0.22 | 1.59 | 0.18 | 1.61 | 0.16 | 1.76 | 0.18 |
| Surprise | 1.53 | 0.17 | 2.14 | 0.19 | 2.69 | 0.25 | 3.70 | 0.36 | 1.52 | 0.19 | 1.91 | 0.18 | 2.66 | 0.26 | 3.44 | 0.37 |
| Threat | 1.50 | 0.16 | 2.56 | 0.23 | 4.09 | 0.29 | 5.66 | 0.32 | 1.33 | 0.14 | 2.14 | 0.20 | 3.63 | 0.33 | 5.02 | 0.39 |
