# Supplementary material for: Self-Relevance Appraisal Influences Facial Reactions to Emotional Body Expressions
Source: PLoS One. 2013 Feb 6;8(2):e55885. doi: 10.1371/journal.pone.0055885 (PMC3566069; doi:10.1371/journal.pone.0055885)
Supplement: Table S3 — Mean (SEM) data from the Corrugator activity submitted to a repeated measures ANOVA using Target of Attention (Self or Other), Level of Emotion (1, 2, 3, 4) and Time Windows (10) as within-subject factors. (DOC) [file pone.0055885.s003.doc]

|  | Self | | | | | | | | Other | | | | | | | |
| --- | --- | --- | --- | --- | --- | --- | --- | --- | --- | --- | --- | --- | --- | --- | --- | --- |
|  | Level1 | | Level2 | | Level3 | | Level4 | | Level1 | | Level2 | | Level3 | | Level4 | |
|  | Mean | SEM | Mean | SEM | Mean | SEM | Mean | SEM | Mean | SEM | Mean | SEM | Mean | SEM | Mean | SEM |
| 100 ms | .075 | .055 | .149 | .052 | .125 | .054 | .184 | .045 | .118 | .040 | .035 | .047 | .038 | .052 | .050 | .054 |
| 200 ms | .160 | .078 | .303 | .067 | .287 | .076 | .364 | .047 | .186 | .057 | .085 | .070 | .233 | .065 | .110 | .067 |
| 300 ms | .269 | .084 | .482 | .070 | .451 | .074 | .649 | .051 | .350 | .078 | .248 | .092 | .374 | .078 | .198 | .078 |
| 400 ms | .374 | .086 | .664 | .073 | .676 | .085 | .859 | .054 | .468 | .074 | .397 | .098 | .411 | .093 | .288 | .082 |
| 500 ms | .434 | .085 | .753 | .073 | .745 | .087 | .969 | .061 | .508 | .080 | .496 | .095 | .459 | .089 | .333 | .081 |
| 600 ms | .453 | .091 | .734 | .076 | .742 | .085 | .976 | .076 | .524 | .088 | .578 | .101 | .450 | .091 | .434 | .082 |
| 700 ms | .429 | .096 | .656 | .078 | .734 | .093 | .964 | .088 | .484 | .085 | .649 | .095 | .458 | .107 | .509 | .085 |
| 800 ms | .449 | .100 | .658 | .082 | .675 | .095 | .857 | .090 | .398 | .070 | .641 | .084 | .468 | .105 | .487 | .081 |
| 900 ms | .449 | .102 | .635 | .082 | .630 | .096 | .803 | .095 | .406 | .079 | .677 | .088 | .472 | .105 | .422 | .083 |
| 1000 ms | .457 | .099 | .620 | .090 | .596 | .100 | .731 | .102 | .366 | .087 | .726 | .100 | .426 | .107 | .411 | .092 |
