# Supplementary material for: Self-Relevance Appraisal Influences Facial Reactions to Emotional Body Expressions
Source: PLoS One. 2013 Feb 6;8(2):e55885. doi: 10.1371/journal.pone.0055885 (PMC3566069; doi:10.1371/journal.pone.0055885)
Supplement: Table S4 — Mean activity (SEM) between 300 and 700 ms for the Corrugator muscle region submitted to a repeated measures ANOVA with within-subject factors of Target of Attention (Self or Other) and Level of Emotion (1, 2, 3, 4). (DOC) [file pone.0055885.s004.doc]

|  | Self | | | | | | | | | | | | Other | | | | | | | | | |
| --- | --- | --- | --- | --- | --- | --- | --- | --- | --- | --- | --- | --- | --- | --- | --- | --- | --- | --- | --- | --- | --- | --- |
|  | Level1 | | | Level2 | | | Level3 | | | Level4 | | | Level1 | | | Level2 | | | Level3 | | | Level4 |
| Mean | .391 | | | .658 | | | .669 | | | .883 | | | .467 | | | .473 | | | .430 | | | .352 |
| SEM | .079 | | | .062 | | | .073 | | | .056 | | | .072 | | | .088 | | | .082 | | | .074 |
|  |  |  |  | |  |  | |  |  | |  |  | |  |  | |  |  | |  |  | |
