# Supplementary material for: Self-Relevance Appraisal Influences Facial Reactions to Emotional Body Expressions
Source: PLoS One. 2013 Feb 6;8(2):e55885. doi: 10.1371/journal.pone.0055885 (PMC3566069; doi:10.1371/journal.pone.0055885)
Supplement: Table S5 — Mean (SEM) data from the zygomatic activity submitted to a repeated measures ANOVA using Target of Attention (Self or Other), Level of Emotion (1, 2, 3, 4) and Time Windows (10) as within-subject factors. (DOC) [file pone.0055885.s005.doc]

|  | Self | | | | | | | | Other | | | | | | | |
| --- | --- | --- | --- | --- | --- | --- | --- | --- | --- | --- | --- | --- | --- | --- | --- | --- |
|  | Level1 | | Level2 | | Level3 | | Level4 | | Level1 | | Level2 | | Level3 | | Level4 | |
|  | Mean | SEM | Mean | SEM | Mean | SEM | Mean | SEM | Mean | SEM | Mean | SEM | Mean | SEM | Mean | SEM |
| 100 ms | -.004 | .046 | .030 | .044 | .038 | .045 | .004 | .053 | .044 | .048 | .025 | .043 | .017 | .041 | -.033 | .052 |
| 200 ms | .012 | .064 | .072 | .062 | .063 | .069 | -.010 | .066 | .049 | .059 | .022 | .068 | .030 | .069 | -.011 | .068 |
| 300 ms | .040 | .077 | .040 | .079 | .102 | .078 | -.017 | .073 | .035 | .069 | .039 | .075 | .011 | .093 | .017 | .079 |
| 400 ms | .071 | .084 | .067 | .094 | .160 | .090 | -.010 | .074 | .048 | .077 | .077 | .079 | .009 | .106 | -.012 | .078 |
| 500 ms | .096 | .089 | .085 | .104 | .173 | .103 | .016 | .079 | .005 | .081 | .082 | .088 | .003 | .104 | -.012 | .086 |
| 600 ms | .040 | .091 | .054 | .106 | .133 | .106 | .025 | .081 | -.056 | .084 | .085 | .091 | -.070 | .094 | -.036 | .080 |
| 700 ms | .030 | .094 | .054 | .113 | .179 | .108 | .057 | .077 | -.044 | .091 | .059 | .087 | -.092 | .087 | -.055 | .081 |
| 800 ms | -.016 | .109 | .049 | .102 | .184 | .102 | .060 | .081 | -.011 | .087 | .051 | .090 | -.079 | .085 | -.036 | .084 |
| 900 ms | .000 | .111 | -.025 | .098 | .149 | .099 | .022 | .087 | -.031 | .090 | .047 | .100 | -.073 | .090 | .024 | .093 |
| 1000 ms | -.033 | .110 | -.035 | .103 | .186 | .101 | .032 | .080 | .022 | .091 | .061 | .099 | -.049 | .082 | .012 | .091 |
